# DeCoRE: A computational method to resolve RNA structural heterogeneity from RNA structure probing data by direct RNA sequencing

**DOI:** 10.1126/sciadv.aef0670

**Published:** 2026-10-02

**Authors:** Yong Huang, Jinsong Zhang, Lei Sun, Qiangfeng Cliff Zhang

**Affiliations:** ^1^ State Key Laboratory of Membrane Biology, MOE Key Laboratory of Bioinformatics, Center for Synthetic and Systems Biology, Beijing Advanced Innovation Center for Structural Biology & Frontier Research Center for Biological Structure, Tsinghua-Peking Center for Life Sciences, Center for Life Sciences and Artificial intelligence, School of Life Sciences, Tsinghua University, Beijing 100084, China.; ^2^ State Key Laboratory of Pathogen and Biosecurity, Academy of Military Medical Sciences, Beijing 100071, China.; ^3^ Shandong Provincial Key Laboratory of Animal Cell and Developmental Biology, School of Life Sciences, Shandong University, Qingdao 266237, China.; ^4^ State Key Laboratory of Microbial Technology, Shandong University, Qingdao 266237, China.

## Abstract

RNA molecules are inherently dynamic, forming complex structural ensembles of alternative conformations that direct their cellular functions. Current methods for resolving RNA structural heterogeneity and dynamics have been developed for short-read sequencing–based structure probing, limiting their sensitivity and resolution. Here, we introduce DeCoRE (deconvolving complex RNA structural ensembles), a computational method that leverages direct RNA sequencing to resolve RNA structural heterogeneity and dynamics at the individual transcript level. DeCoRE combines enhanced structural signal detection with direct clustering of sequencing reads to sensitively deconvolve RNA structural ensembles at single-nucleotide resolution. We demonstrate DeCoRE’s capabilities by resolving the known alternative conformations of the lysine and TPP riboswitches and uncover a previously uncharacterized cotranscriptional folding pathway of bacterial ribosomal RNA, where two conformations coexist during early elongation and progressively converge into a single mature conformation. DeCoRE represents a powerful tool for dissecting RNA structural heterogeneity and dynamics, advancing our understanding of RNA function.

## INTRODUCTION

RNA molecules form complex structures that are integral to their catalytic and regulatory roles in biological processes (*1*). These structures are inherently dynamic, adopting alternative conformations that facilitate functional plasticity (*2*–*4*). For instance, the *Bacillus subtilis* lysine riboswitch was shown to undergo ligand-dependent shifts, toggling between so-called terminator and antiterminator helices to regulate transcription termination (*5*). Similarly, ribosomal RNA (rRNA) exhibits transient, dynamic association with ribosomal proteins, forming heterogeneous conformations during transcription that guide the ribosome assembly—a process critical for ribosome biogenesis (*6*, *7*).

Over the past decade, high-throughput RNA structure probing technologies have been developed to enable transcriptome-wide profiling of RNA structures across diverse biological contexts (*1*, *8*). These methods use chemicals such as dimethyl sulfate (DMS) and SHAPE reagents (e.g., NAI) to selectively modify unpaired or flexible RNA nucleotides, with structural information inferred from modification-induced reverse transcription artifacts of truncated cDNAs or mutations, detected via deep sequencing (*9*). However, these approaches only report average structures for RNA structural ensembles, obscuring their inherent conformational heterogeneity. Computational tools such as RING-MaP (*10*), DREEM (*11*), DRACO (*12*), and DANCE-MaP (*13*), have been developed to resolve RNA structural heterogeneity, extracting distinct conformations by analyzing comutation patterns within sequencing reads from RNA structure probing experiments. Despite their utility, these methods are designed for probing technologies based on short-read sequencing platforms, and therefore their sensitivity and resolution to resolve structural heterogeneity is inherently limited by read-length constraints.

Recent advances in RNA structure probing have extended to long-read sequencing platforms, including direct RNA sequencing (DRS)–based methods [e.g., PORE-Cupine (*14*) and nanoSHAPE (*15*)] and cDNA sequencing–based approaches [e.g., Nano-DMS-MaP (*16*) and smStructure-seq (*17*)]. While these techniques have enabled the determination of RNA structures at their full length, it remains challenging to resolve structural heterogeneity within individual transcripts, owing to sparse structural signals resulting from the low modification rate in structure probing experiments. For example, the DaVinci algorithm (*17*), used in smStructure-seq, first predicts a structure model for each read based on its mutation profile and then clusters the models to identify RNA structural conformations. However, the sparse structural information per read has compromised the accuracy in obtaining RNA structures models (*18*), which may lead to inaccurate resolution of alternative conformations.

Here, we present DeCoRE, a computational method to deconvolve complex RNA structural ensembles from DRS-based RNA structure probing data (https://github.com/huangyon20/DeCoRe; fig. S1A). DeCoRE advances RNA structure analysis through two innovations: (1) enhanced structural signal detection by incorporating miscalled base, (2) resolution of alternative conformations via direct clustering of structurally distinct reads. Our results showed that DeCoRE achieves high accuracy in resolving RNA structural heterogeneity and dynamics at the individual transcript level, as evidenced in studies resolving known RNA structural heterogeneity including two riboswitches. Last, we designed an experimental investigation and used DeCoRE to uncover a dynamic cotranscriptional folding pathway in 16*S* rRNA transcription, revealing early structural plasticity with two distinct conformations converging into a single conformation through 3′ end rearrangements during transcription. DeCoRE stands as an effective tool, poised to deepen our understanding of RNA dynamic structures and function across diverse biological systems.

## RESULTS

### DeCoRE enhances RNA structural information retrieval from DRS probing data

DRS analysis determines the nucleotides (basecalling) of an RNA molecule by measuring characteristic electrical current changes as the RNA threads pass through nanopores. For RNA structure probing applications, machine learning is used to analyze current signal shifts with respect to the reference of an unmodified control, categorizing basecalling outcomes as (1) matched bases that are identical to the reference, (2) modified bases exhibiting characteristic probing-induced signal shifts, or (3) miscalled bases, where substantial deviations lead to identification as a different nucleotide (fig. S1B). Given the present high error rate of DRS, miscalled bases are excluded from structural analyses as they cannot be reliably distinguished from basecalling errors (*19*).

Intriguingly, in a public RNA structure probing dataset (*15*), we observed moderate correlations between miscalled base rate against modified base rate in Aclm-treated RNA and the difference (ΔMis_R) in miscalled bases between Aclm-treated and dimethyl sulfoxide (DMSO)–treated reads (Pearson ρ = 0.36 and 0.53, respectively; fig. S1C), showing that miscalled bases reflect structural information similarly as with modified bases. Notably, miscalled bases occurred at a median rate of 9.6% per position, far exceeding the rate of 2.1% observed for modified bases. This finding highlights their high potential to enhance structural information obtainable in RNA structure probing by direct sequencing.

DeCoRE is designed to harness this untapped information in DRS data by integrating information for both modified bases (probing-induced changes) and miscalled bases (“sequencing errors” reflecting structural constraints) (Fig. 1A and Materials and Methods). We used unsupervised anomaly detection (UAD) algorithms to identify structural signals. We tested the performance of various UAD algorithms and input feature combinations based on agreement between calculated reactivity scores and known structure models for six pri-miR-17∼92a RNA elements from a public dataset (*15*). One-class support vector machine (SVM) model and the using of all three current signals as input of the SVM model (i.e., mean, SD, and dwell time of the signals) outperforms the other alternatives (fig. S1D). We found that incorporating both modified bases and miscalled bases, using a one-class SVM model and three current signals, yielded the highest agreement with known structural models (AUC = 0.820) (Fig. 1B). Notably, using only miscalled bases in 150 nM Aclm-treated RNA also yielded reasonably high agreement (AUC = 0.706), confirming that miscalled bases reflect RNA structural information, and this accuracy could be further improved by taking DMSO-treated libraries as a control (AUC = 0.779). These results were repeatable on the other two datasets (75 and 100 nM Aclm-treated RNA; fig. S1E). Then, we performed downsampling of the reads and evaluated structural prediction performance across different coverage levels. The integration of ΔMis_R consistently outperforms the use of modified bases alone (fig. S1F), with the advantage becoming more pronounced at lower sequencing depths.

**Fig. 1.**
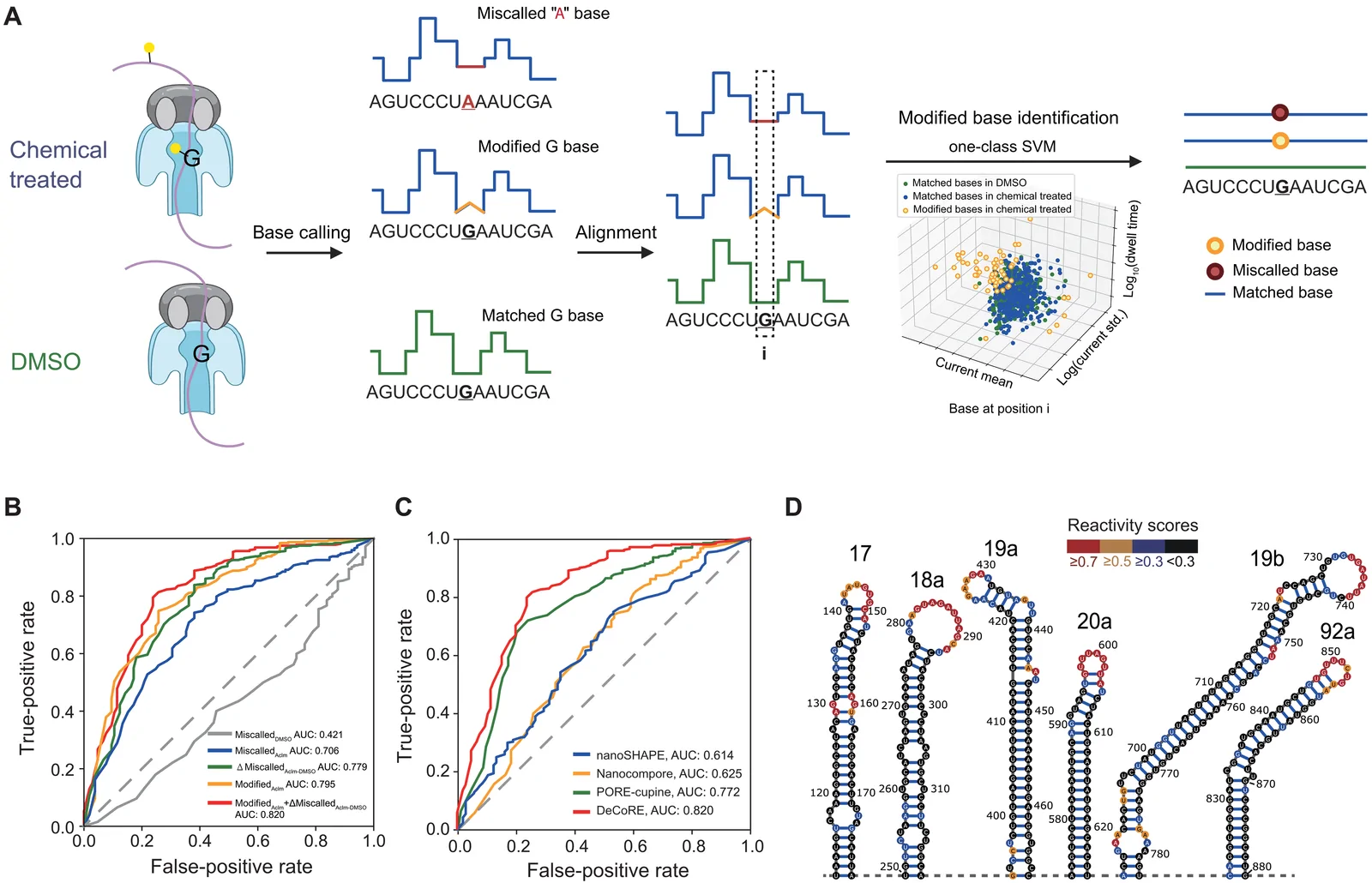
DeCoRE retrieves accurate structural information using enhanced structural profile. (**A**) Schematic overview of DeCoRE for extracting structural information from nanopore sequencing data. In structure probing experiments, RNA was treated with structure probing chemicals (e.g., NAI-N3) or control reagent (DMSO) and sequenced using nanopore DRS. Then, DeCoRE aligned the reads together with their raw current signals to the reference RNA. For each nucleotide along the reference, a three-feature one-class SVM model was used to identify current signal anomalies in the chemical treated reads by comparing the matched bases, which were subsequently designated as modified bases. The resulting enhanced structural profile of the chemical-treated reads integrates both miscalled bases and modified bases. (**B**) Performance evaluation of structural profile incorporating both miscalled bases and modified bases in 150 mM Aclm-treated RNA. ROC curves analysis demonstrates strong agreement with known structural models of pri-miR-17∼92a RNA. Using only miscalled bases in Aclm-treated RNA yielded reasonably high agreement with the structural models. (**C**) ROC curves of reactivity scores calculated by DeCoRE and other methods (Materials and Methods), evaluated on known structural models of pri-miR-17∼92a RNA. (**D**) The six known structures of pri-miR-17∼92a RNA colored with the reactivity scores calculated by DeCoRE.

We benchmarked DeCoRE against three existing methods [i.e., PORE-Cupine (*14*), nanoSHAPE (*15*), and Nanocompore (*20*)] (table S1) using their default parameters. Through comparative analysis of AUC and AUPRC curves, DeCoRE outperformed alternatives in agreement with known pri-miR-17∼92a RNA structural models (Fig. 1, C and D, and fig. S1G), demonstrating its ability to effectively extract structural information from DRS data by leveraging previously overlooked miscalled bases. The basecalling algorithms/models directly affects the frequency of miscalled bases. We basecalled the raw FAST5 data using the current mainstream basecaller Guppy (V6.5.7) and Dorado (v0.8.2) and recalculated for a highly consistent AUC values (fig. S1H), demonstrating the stability of DecoRE across different basecalling strategies.

### DeCoRE resolves known and previously uncharacterized RNA structural heterogeneity

Building from the enhanced RNA structural information detection based on incorporation of miscalled bases, DeCoRE addresses the challenge of resolving RNA structural heterogeneity through an innovative clustering approach (Fig. 2A and Materials and Methods). Specifically, DeCoRE first encodes each sequencing read into a vector, respectively representing matched, modified, and miscalled bases as 0, 1, and 2. It then clusters the vectors and calculates a “reactivity score profile” for each group. Last, DeCoRE uses the RNA structure prediction tool, RNAfold (*21*), to build cluster-specific models, constrained by reactivity scores (*22*).

**Fig. 2.**
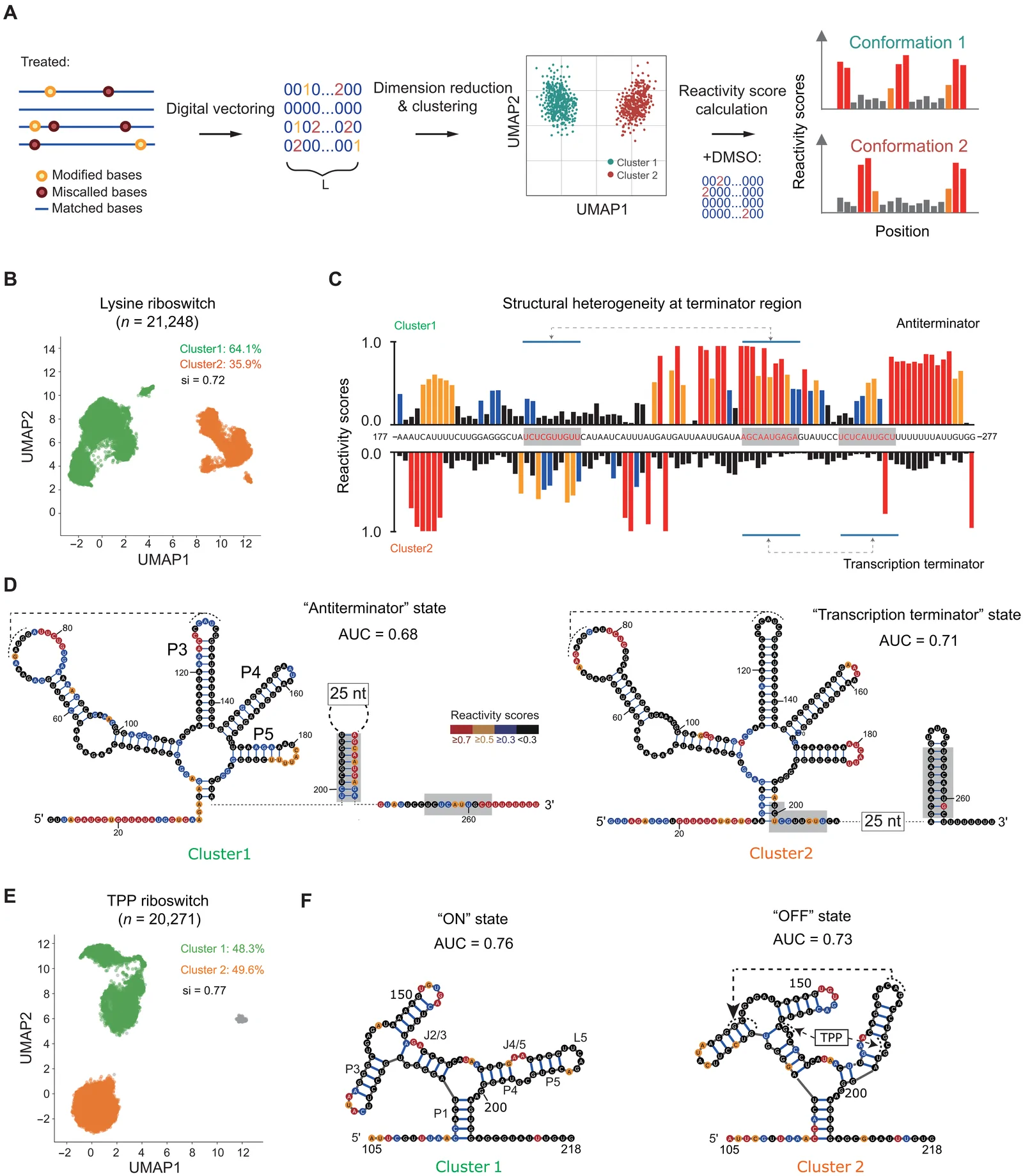
DeCoRE identifies the known heterogenous structures of riboswitches. (**A**) Schematic of the clustering approach used to resolve heterogenous structures from enhanced structural profiles. Each read’s structural profile was encoded as a vector, where bases were categorized as 0 (matched bases, blue), 1 (modified bases, light blue), or 2 (miscalled bases, red). All vectors were padding to the same length (L) corresponding to the reference RNA. (**B**) Uniform Manifold Approximation and Projection (UMAP) of the vectorized structural profile of each read in chemical-treated sample of the lysine riboswitch, colored by the cluster identified by DeCoRE. The silhouette score (si) was used to assess the clustering quality. The relative proportions of each cluster were indicated. (**C**) Barplots displayed the reactivity scores of the two structural cluster identified by DeCoRE in the region harboring the two mutually exclusive conformations of the lysine riboswitch—the antiterminator and transcription terminator states. The three key regions distinguishing the two conformations are highlighted by gray boxes and blue lines, and the base-pairing patterns in these regions for the two conformations was indicated by dashed lines. (**D**) The two structural models corresponding to the antiterminator and transcription terminator states were colored with the DeCoRE’s cluster-specific reactivity scores. (**E**) UMAP of the vectorized structural profile of each read in chemical-treated sample of the TPP riboswitch, colored by the cluster identified by DeCoRE. The relative proportions of each cluster were indicated. (**F**) The structure model of TPP riboswitch aptamer region (THI element) under ON and OFF state (*24*), colored with the DeCoRE’s cluster-specific reactivity scores. In the TPP-bound OFF state, J4/5 and J2/3 stack onto the TPP ligand, and loop L5 rearranges to form hydrogen bonds with P3, in contrast to the ON state.

We applied DeCoRE to a public RNA structure probing dataset generated by PORE-Cupine (*14*), a nanopore DRS–based method, for two riboswitches with well-characterized conformational switching: the lysine riboswitch, for which ligand binding induces a terminator hairpin formation while ligand release induces an antiterminator conformation that permit transcriptions (*5*), and the thiamine pyrophosphate (TPP) riboswitch, which regulates gene expression through aptamer domain rearrangements between “ON” and “OFF” states (*23*, *24*). For the lysine riboswitch, DeCoRE obtained two distinct clusters (64.1 and 35.9% of the total reads, silhouette score si = 0.72) (Fig. 2B). Reactivity profiles well-matched known conformations for each cluster (AUC = 0.68 and 0.71), particularly capturing ligand-dependent rearrangements in the terminator region (Fig. 2, C and D). Similarly, for the TPP riboswitch, DeCoRE identified two clusters (48.3 and 49.6% of the reads si = 0.77) (Fig. 2E), with reactivity profiles again aligning well with known aptamer region conformations (AUC = 0.76 and 0.73) (Fig. 2F).

Notably, excluding miscalled bases resulted in all sequencing reads collapsing into a single cluster for both riboswitches (fig. S2, A and B), underscoring the critical role of these miscalled bases in accurately resolving conformational heterogeneity. In comparison, the DaVinci algorithm also identified two clusters for both riboswitches, yet its structural models aligned poorly with the known structure conformations (AUC = 0.52 and 0.65 for the lysine riboswitch, and AUC = 0.49 and 0.45 for the TPP riboswitch) (fig. S2, C to F). These results highlight DeCoRE’s ability to resolve RNA structural heterogeneity by harnessing sequencing “noise” (miscalled bases) as structure signals and applying innovative clustering, capturing biologically meaningful conformational states.

We further applied DeCoRE to another public PORE-Cupine dataset targeting the subgenomic RNAs (sgRNAs) of severe acute respiratory syndrome coronavirus 2 (SARS-CoV-2) (*25*), in which study only an averaged representative structure is reported for each RNA. Focusing on sgRNA N (the most highly expressed sgRNA) (fig. S3A and Materials and Methods), DeCoRE identified two distinct conformational clusters, with a dominant cluster comprising 81.6% of total reads (fig. S3B). With exclusion of the miscalled bases, the reads collapsed into a single cluster, demonstrating that miscalled bases play a critical role in resolving structural heterogeneity. The reactivity scores of the two clusters were calculated separately, and their differences mainly localized to the 3′ untranslated region (3′UTR) of sgRNA N (fig. S3C). Notably, the original study associated with this dataset provides only an averaged structural model for the SARS-CoV-2 3′UTR, without resolving genomic/sgRNA and their structural heterogeneity. We compared the two cluster-specific structures identified by DeCoRE with the reported 3′UTR model (fig. S3D). For several local structural elements in the averaged model, the two clusters exhibit complementary reactivity patterns, with one showing high scores where the other is low. This observation indicates that the averaged structure does not fully capture the underlying conformational heterogeneity. These findings highlight DeCoRE’s ability to uncover previously unrecognized structural heterogeneity.

### DeCoRE uncovers structural transitions during 16*S* rRNA transcription at single-nucleotide resolution

While the structure of rRNA is well characterized in its mature state, how it undergoes dynamic structural changes during transcription has only been considered empirically but remains much less well studied (*6*). To investigate this, we generated a series of transcripts mimicking transcription intermediates of *B. subtilis* 16*S* rRNA and profiled their structures using a DRS-based RNA structure probing protocol slightly modified from PORE-Cupine (Fig. 3A, fig. S4, and Materials and Methods) (*14*). In brief, we amplified *B. subtilis* 16*S* DNA using T7 promoter–ligated primers and then randomly digested it with deoxyribonuclease I (DNase I) to generate a mixture of diverse DNA fragments. This mixture was subsequently in vitro transcribed, yielding transcripts sharing a common 5′ end but varying in 3′ ends. The resulting transcripts were modified using NAI-N3 or DMSO and sequenced using nanopore DRS.

**Fig. 3.**
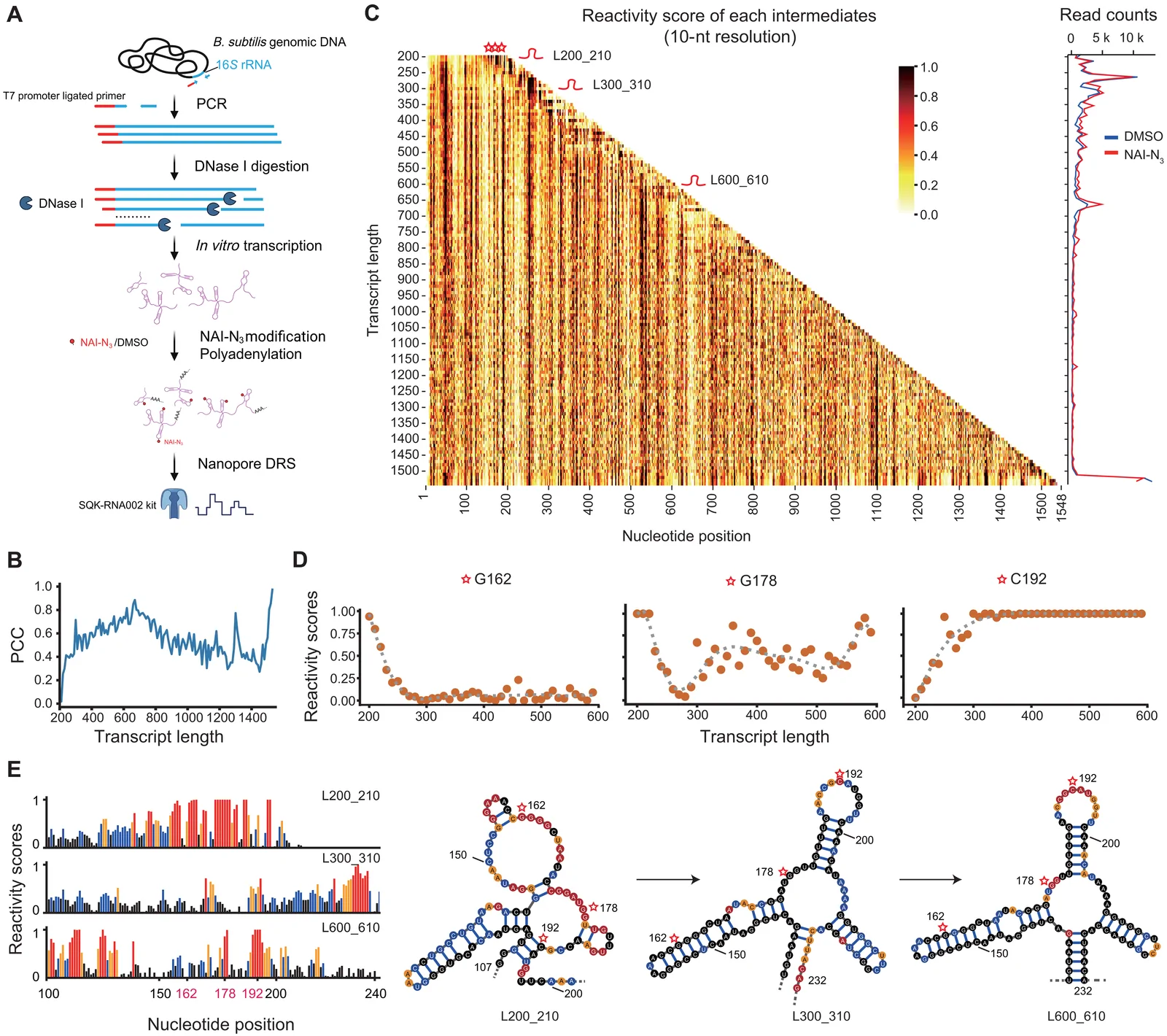
DeCoRE reveals structural transitions during *B. subtilis* 16*S* rRNA transcription. (**A**) Schematic overview of the experimental workflow used to generate the transcriptional intermediates of *B. subtilis* 16*S* rRNA in vitro and detect their structures using nanopore DRS–based structure probing method. (**B**) PCC of reactivity scores between each intermediate and the full-length 16*S* rRNA within their shared nucleotide regions. (**C**) Left heatmap showed the reactivity scores of 136 intermediates, categorized on the basis of their 3′ end alignment positions with 10-nt resolution. Each row represents the reactivity scores of a specific intermediate with length indicated by the *y* axis. Each column represents the reactivity score trajectories for a specific nucleotide, indicated along the *x* axis. Nucleotides highlighted with asterisk were examples [analyzed in (D)], which exhibit substantial reactivity score variation across different intermediates. Right line plot showed the read count distribution of each transcriptional intermediate. (**D**) Reactivity trajectories of three representative residues, corresponding to the highlighted nucleotides in (B), illustrate distinct structural transition patterns: G162 exhibits early base-pairing (“immediate pairing”), C192 remains unpaired across intermediates (“immediate unpaired”), and G178 shows mixed reactivity indicative of dynamic structural shifts. (**E**) Reactivity score profiles and predicted structure models of the selected intermediates of lengths 200 to 210 nt (L200_210), 300 to 310 nt (L300_310), and 600 to 610 nt (L600_610). The structure model of each intermediate was predicted using their all nucleotides with the reactivity scores as constraints.

We mapped the sequencing reads to the *B. subtilis* 16*S* rRNA reference and clustered them into 136 groups, according to the 3′ positions and with a 10–nucleotide (nt) resolution, and finally calculated the reactivity scores for each group (Materials and Methods). For clarity, we designate these transcript groups as *B. subtilis* 16*S* rRNA “intermediates,” denoted by their 3′ end positions. For instance, intermediate L200_210 refers to the group of transcripts with 3′ ends mapping within the 200- to 210-nt range. We observed high reproducibility of reactivity scores between the two biological replicates for both full-length and intermediate 16*S* rRNAs (e.g., *R* = 0.96 for the full-length 16*S* rRNA, *R* = 0.59 to 0.95 for the intermediates) (fig. S4, E and F). Moreover, the full-length reactivity scores correlated well with the established structural model from RNAcentral (AUC = 0.69) (fig. S4G) (*26*).

We compared the structures of intermediate rRNAs with the full-length rRNA by calculating Pearson correlation coefficients (PCCs) for reactivity scores of shared regions in each comparison (Fig. 3B). In contrast to replicate samples, we found low PCCs between intermediates and full-length RNA, indicating that 16*S* rRNA intermediates forms structures distinct to the final mature conformation.

We traced and analyzed the reactivity score trajectories at single-nucleotide resolution (each column of the matrix in Fig. 3C). First of all, we noted structural transitions across most nucleotides. Briefly, we used a sliding window to scan each nucleotide’s reactivity score trajectory, identifying transitions when the reactivity scores in the first and second halves of the window showed substantial differences (Materials and Methods). This analysis revealed structural transitions at 84% (1301 of 1548) positions, aligning with a recent report that most (∼77%) nucleotides in nascent pre-rRNA undergo substantial structural changes during transcription (*27*).

Further, we identified distinct patterns of structural transitions for individual nucleotides. For example, G162 underwent a gradual transition from unpaired to paired states, while C192 shifted from paired to unpaired. In contrast, G178 exhibited multiple transitions between paired and unpaired states throughout the folding process (Fig. 3D). To visualize these structural rearrangements, we selected three representative intermediates (specific rows of the matrix in Fig. 3C) and modeled their secondary structures using reactivity scores as constraints (Fig. 3E). We observed that G162 initially resided within a large internal loop that progressively formed base pairs, ultimately reorganizing into an extended stem structure. Conversely, C192 began in a stem region that subsequently melted to form a loop in a big stem-loop structure. These results reveal the prevalence and diversity of local RNA structure transitions during transcription, highlighting the highly dynamic nature of rRNA folding.

### DeCoRE uncovers structural conformational dynamics during 16*S* rRNA transcription

We selected five intermediates with high (≥4000×) sequencing coverage, ranging from 270 to 1540 nt and representing distinct, progressive stages of RNA synthesis, to dissect structural conformational dynamics of cotranscriptional folding. Upon applying DeCoRE to each intermediate, we made a notable observation: The first three intermediates (earlier transcription stages) each resolved into two discrete structural conformations, whereas the last two intermediates (later transcription stages) each adopted only a single conformation (Fig. 4A).

**Fig. 4.**
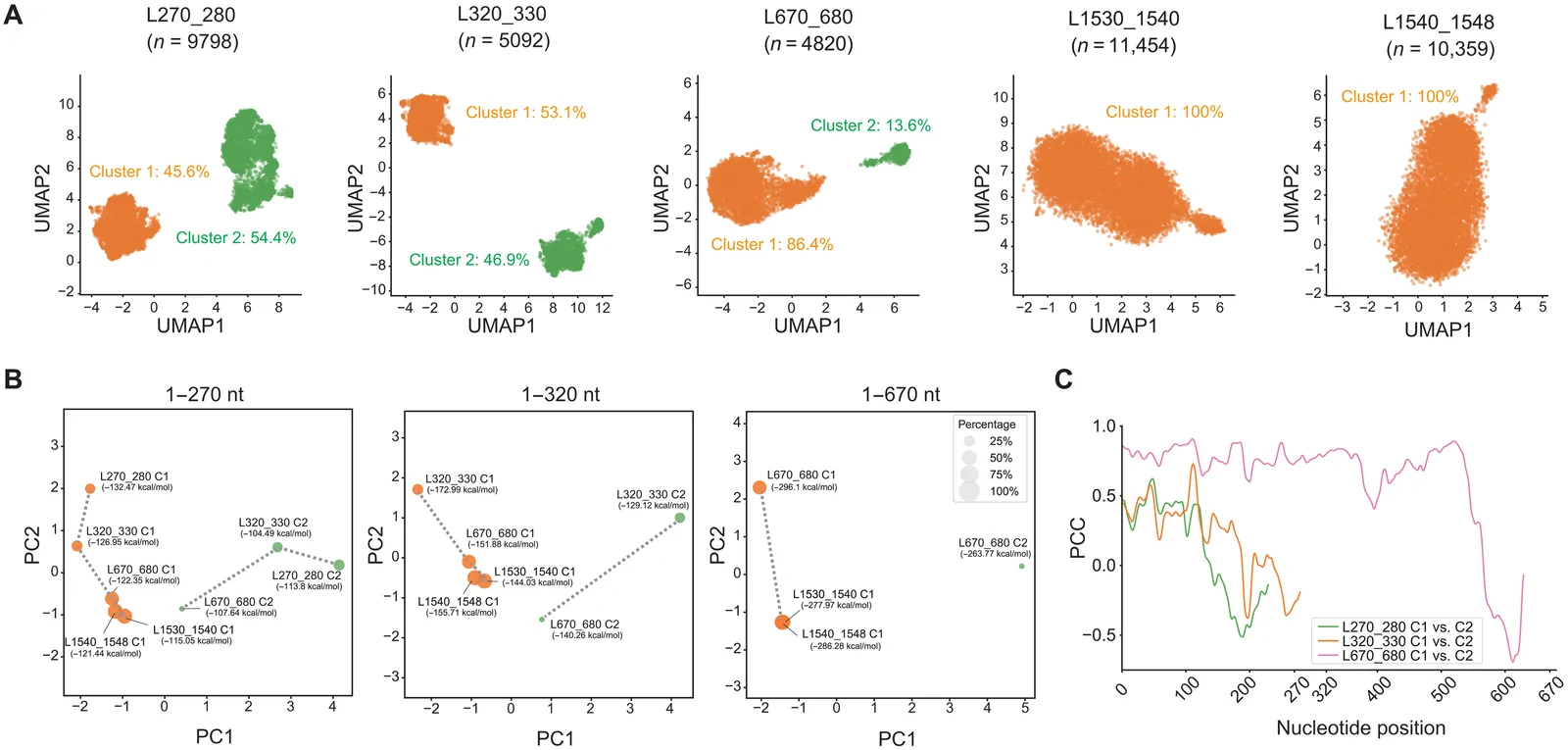
DeCoRE uncovers structural heterogeneity and dynamic folding of *B. subtilis* 16*S* rRNA during the transcription. (**A**) UMAP of the vectorized structural profile of each read in chemical-treated sample of the five 16*S* rRNA transcriptional intermediates with high coverage, colored by the cluster identified by DeCoRE. The relative proportions of each cluster were indicated. (**B**) Scatter plot of the PCA analysis of reactivity scores for the 1- to 270-nt, 1- to 320-nt, and 1- to 670-nt regions of each conformation across all five intermediates. Each dot represents a conformation that predicted within the corresponding region, labeled with structural model free energy (kcal/mol). Dot colors indicate conformation category, and dots belonging to the same structurally related group are connected by dashed lines. Dot size reflects the relative abundance of each conformation within the corresponding transcription intermediates. (**C**) PCC of reactivity scores between the two conformations for each of the three intermediates: L270_280, L320_330, and L670_680, calculated using a 50-nt sliding window.

To explore potential relationships among these conformations, we calculated reactivity scores for each conformation and assessed their structural similarities using pairwise PCC of reactivity scores across shared regions (Materials and Methods). This analysis revealed a correspondence among all observed conformations, categorizing them into two structurally related groups that we designated “Conformation 1” (C1) and “Conformation 2” (C2) (fig. S5A). Principal components analysis (PCA) on the reactivity score profiles of each conformation across all five intermediates confirmed this correspondence and further revealed a detailed convergence trajectory: as transcription proceeds, C1 and C2 gradually approach each other, with C1 becoming increasingly predominant at later transcription stage (Fig. 4B). This trend was observed for shared regions of different lengths of all cases including the 1- to 270-nt, 1- to 320-nt, and 1- to 670-nt segments. Notably, conformation C1 exhibited greater thermodynamic stability than C2 across different intermediates.

To pinpoint the basis for this dynamic structural convergence, we conducted local structural comparisons using a 50-nt sliding window, identifying regions of low correlation between C1 and C2 primarily at the 3′ terminus of each intermediate (Fig. 4C and Materials and Methods). This suggests that the observed structural heterogeneity stems mainly from the thermodynamic instability of the newly transcribed nucleotides at the elongating 3′ terminus. We found that, as the transcript elongates, the structural similarity between the two conformations in the remaining regions (excluding the 3′ termini) also increases, corroborating the above discovery of the convergence of the two conformations (Fig. 4B). Together, these results reveal a previously uncharacterized cotranscription folding pathway for bacterial rRNA, in which two conformations coexist during early transcript elongation and undergo progressive convergence, driven by 3′ terminus folding, to ultimately form a single dominant mature conformation in the final stage.

## DISCUSSION

DeCoRE advances the resolution of RNA structural heterogeneity and dynamics at the individual transcript level from DRS-based structure probing data by synergistically incorporating miscalled bases to enhance structural signal detection and direct clustering of raw sequencing reads to improve resolution of conformational ensembles. Notably, while miscalled bases have been leveraged for identifying endogenous RNA modification in DRS data (*19*), prior DRS-based structure probing analyses have not exploited this rich source of structural information (which is obtained by analyzing exogenous RNA modifications). Our analyses demonstrated that miscalled bases not only occur at a high frequency but also correlate strongly with modification events, thus greatly enhancing structural signals available for analysis. By combining these data streams, DeCoRE outperforms existing DRS-based structure probing pipelines in reconstructing known and previously unidentified RNA conformations.

Another key advance is DeCoRE’s direct clustering strategy, which circumvents a limitation of prior approaches such as DaVinci (*17*). Rather than first predicting per-read structures from sparse modification profiles—an approach inherently sensitive to errors and biases in structure prediction algorithms—DeCoRE clusters reads directly on the basis of their raw digital structural signatures. This approach preserves the full spectrum of observed conformational diversity and avoids information loss and model bias that can arise from premature structure assignment. As demonstrated in our benchmarking analyses, this strategy enables DeCoRE to resolve biologically meaningful conformational states that align with established models for riboswitches.

By leveraging the long-read capabilities of DRS, DeCoRE enables assignment of structures to full-length and intermediate transcripts, providing insights into the dynamic landscape of cotranscriptional RNA folding. Our application of DeCoRE to *B. subtilis* 16*S* rRNA transcription reveals a dynamic pathway characterized by structural plasticity in early transcriptional stages—where two distinct conformations coexist and are progressively resolved into a single, thermodynamically stable mature fold through 3′ end rearrangements. This finding not only echoes but extends previous observations from short read–based probing studies (*27*, *28*), which were limited by their inability to track long-range folding and assign structures to specific transcript intermediates. DeCoRE fills this gap, revealing widespread, position-specific structural transitions across the rRNA length.

The dynamic conformational convergence observed for 16*S* rRNA likely underpins the ability of nascent rRNA to interact with diverse assembly factors and chaperones, facilitating the highly orchestrated process of ribosome biogenesis (*7*). Our results complement and expand on prior studies showing transient association of chaperones and ribosomal proteins with pre-rRNA immediately after synthesis, supporting the observed structural plasticity as a prerequisite for the stepwise assembly and maturation of functional ribosomal particles (*6*, *29*).

In summary, by harnessing the full potential of DRS-based structure probing data, DeCoRE provides a powerful and generalizable platform for dissecting RNA structural heterogeneity and conformational dynamics at unprecedented resolution, opening avenues for investigating the structural basis of RNA function, regulation, and evolution across diverse biological systems. DeCoRE’s approach assumes that miscalled bases reflect structural constraints. However, it is plausible that some of these may arise from sequencing errors unrelated to RNA structure. This could reduce DeCoRE’s accuracy in reads classifying whether the sequencing error rate is high. Notably, newer Nanopore flow cells (e.g., R10.4) offer substantial improvements in basecalling accuracy, which would be expected to enhance DeCoRE’s performance by reducing the noise from nonstructural sequencing errors. Further, we generated a set of *B. subtilis* 16*S* rRNA transcripts with diverse 3′ends to mimic transcription intermediates. While this system provided insights into RNA structural heterogeneity and dynamics, it may have introduced artifacts and does not likely fully recapitulate the complexities of in vivo transcription, which are known to involve factors like transcription speed, ribosome binding and falling, and the process of transcript elongation and release by RNA binding proteins. Applying DeCoRE to more physiologically representative systems for studying cotranscriptional folding should help validate and ideally extend our understanding of rRNA transcription.

## MATERIALS AND METHODS

### Data processing of nanopore sequencing reads

The raw nanopore sequencing output comprises FAST5 (raw signal) and FASTQ (basecalled using Guppy V4.3.4 and rna_r9.4.1_70bps_fast.cfg model) files. Initially, the FASTQ reads were aligned to the reference RNA using either GraphMap (v0.6.3) (*30*) with the -*a* sg option or Minimap2 (v2.1) (*31*) with --*MD* -*ax* map-ont options. Subsequently, SAMtools was used to retain the mapped reads and generate sorted BAM files. The raw signals in FAST5 files were base-aligned to the reference and converted into events using eventalign module of Nanopolish (v0.14.0) (*32*). Redundant events, which originate from the same read and the same nucleotide, were collapsed into single event by calculating the average values of event_level_mean (Current_mean), event_stdv (Current_stdv), while summing the event_length (Dwell time). The bases on the read that could not be aligned with the raw signal remain empty in the event file, which were treated as miscall bases. This procedure yielded nonredundant event datasets for both the chemical-treated and DMSO-treated experiments separately.

### Intermediate selection

Reads in the processed event file were categorized into distinct groups according to their mapping positions on reference genome. The start and end mapping positions of each read, included in the event file, were used to divide reads into individual event files, each representing a unique intermediate. To minimize the influence of degraded RNA reads that may not accurately reflect the true transcript length, a 5′-truncation threshold parameter -s was applied to exclude reads that had truncated more than s nucleotides at the 5′ terminus. The length range of intermediates was controlled by an end position parameter -*e* and a resolution parameter -*b*. Reads with last mapping position within [e, e + b] intervals were classified as a discrete intermediate. In the analysis of *B. subtilis* 16*S* rRNA, we used *s* = 30, *b* = 10.

### Modification bases detection

To assess the modification status of each nucleotide on each read generated from chemical-treated experiment, we used a one-class SVM model. This model used three signal features: Current_mean, log(Current_stdv), and log_10_(Dwell time). For each nucleotide in the reference RNA, the signal features from the DMSO-treated reads were used to train the model, which was then used to infer the modification status of each nucleotide in the chemical-treated reads, generating a structural profile for each read. In this profile, a value of −*1* indicates that the base is classified as an outlier, denoted as modified base, while a value of 1 indicates that the base is classified as a normal one, denoted as matched base. The miscalled bases that cannot be mapped to the references were assigned NaN values.

### Clustering of the sequencing reads

For chemical-treated sequencing reads, the structural profile of each read that belongs to a specific RNA transcript was converted into a vector comprising 1 s (modified bases or SVM outliers), 0 s (matched bases), and 2 s (miscalled bases). Each vector that represents one read was padding to length L, with outlying NaN positions filled with zeros. Then, the vectors were subjected to Uniform Manifold Approximation and Projection (UMAP) (*33*) for dimensionality reduction and visualization. Subsequently, vectors were clustered using either Gaussian Mixture or DBSCAN method. Following reads classification, reactivity scores were calculated for each cluster.

### Calculating the reactivity scores

Reactivity scores of an RNA molecule were derived by normalizing modification rates across the RNA transcript. For each nucleotide position “i,” the modification rate was calculated from structural profiles of both chemical-treated and DMSO-treated reads using the following equations

$$\Delta \mathrm{Mis}\_R_{i}={\left(\frac{N_{{\text{miscalled}}_{i}}}{N_{i}}\right)}_{\text{Treated}}-{\left(\frac{N_{\text{mi}{\text{scalled}}_{i}}}{N_{i}}\right)}_{\text{DMSO}}$$ (1)

$$R_{i}={\left(\frac{N_{{\text{modified}}_{i}}}{N_{{\text{modified}}_{i}}+N_{{\text{matched}}_{i}}}\right)}_{\text{Treated}}+\Delta \mathrm{Mis}\_R_{i}$$ (2)

where $N_{{\text{modified}}_{i}}$ represents the counts of modified bases, $N_{{\text{miscalled}}_{i}}$ represents the counts of miscalled bases, and $N_{{\text{matched}}_{i}}$ represents the counts of matched bases at position i along the RNA. $\Delta \mathrm{Mis}\_R_{i}$ was the difference on miscalled bases rate at position i between chemical-treated and DMSO-treated reads. The resulting rates $R_{i}$ were then normalized via Winsorization (*34*) with 200-nt window size and 5-nt steps. Specifically, a sliding window approach was applied across the RNA sequence, setting the top 5% rates to 1 and the bottom 5% rates to 0, while scaling the remaining rates within a range of 0 to 1. The final reactivity score was obtained by averaging the scores $R_{i}$ from all sliding windows.

### Processing of SARS-CoV-2 sgRNAs nanopore sequencing data

Raw FAST5 files were retrieved from the National Center for Biotechnology Information under GEO accession IDs: GSM5024398 and GSM5024399. The Guppy(V4.3.4) basecaller (*35*) was used to convert the FAST5 files into FASTQ sequences with rna_r9.4.1_70bps_fast.cfg model. Subsequently, BLASTn was applied to search the reads against the nine reference sgRNAs in the article (*25*), with the following parameters: -*outfmt* 7, −*num_descriptions* 1, and -*num_alignments* 1. The sgRNA N–assigned reads were filtered with alignment position filtering thresholds to ensure for complete leader sequences and sufficient length. Specifically, reads were selected if their alignment positions met the criteria of *sstart* < 30, *send* > 300. Subsequently, the selected reads were randomly down-sampled to 15,000 for both two datasets and performed the modification bases detection and clustering. The AUC of local structure element was calculated based on the bases in gray boxes.

### Construction of *B. subtilis* 16*S* rRNA transcription intermediates library

Genomic DNA was isolated from *B. subtilis* strain 168 using the TIANamp Bacteria DNA Kit. PCR amplification was carried out to obtain the full-length 16S rRNA DNA template with primers containing the T7 promoter-containing sequence:

Forward primer (F): TAATACGACTCACTATAGTTATCGGAGAGTTTGATCCTGGC

Reverse primer (R): TACACGACGCTCTTCCGATCTAGGAGGTGATCCAGCCGCAC

The amplified DNA was fragmented with Turbo DNase I (Thermo Fisher Scientific, 2 U/μl) at varying concentrations (0.008 to 0.002 U/μl). The optimal DNase I concentration, determined by analyzing the digestion efficiency on an agarose gel, was found to be 0.004 U/μl. Subsequently, in vitro transcription was performed using the HiScribe T7 Quick Kit with the digested DNA fragments following the manufacturer’s protocol. After overnight incubation, residual DNA was removed with DNase I treatment. For RNA chemical modification, NAI-N3 was added to the RNA samples to a final concentration of 100 mM, followed by incubation at 37°C for 10 min. As a control, DMSO was added to a parallel reaction at an equivalent concentration. Last, the modified RNAs were polyadenylated at the 3′ end using the Poly(A) Tailing Kit (Thermo Fisher Scientific, AM1350).

### Nanopore DRS

DRS was performed as described in PORE-cupine (*14*) except that the DRS library preparation was carried out using the SQK-RNA002 kit instead of SQK-RNA001 kit from Oxford Nanopore Technologies. To facilitate reuse of the flow cells, a wash kit (EXP-WSH004) was used for thorough cleaning.

### Processing of *B. subtilis* 16*S* rRNA nanopore sequencing data

The raw FASTQ reads of each experiment were basecalled using Guppy (V4.3.4) and mapped to the reference using Minimap2 (*31*). Some reads that mapped onto the reverse-complementary strand of reference were not the transcribed RNA and were filtered as reverse-mapped reads. Some reads that had a noncomplete 5′ terminal were filtered as 5′ truncated reads. Besides, at the step of event alignment, some reads could not be aligned to their current signal data in FAST5 files and were filtered as no-signal reads.

### Detection of structural transition between transcription intermediates

To identify structural transitions between transcription intermediates as transcripts extend, a sliding window with a length of 10 (spanning 100 nts) and step size of 1 was used to scan the reactivity scores trajectory in Fig. 3C for each nucleotide. Only windows with valid reactivity scores in all positions were considered. Structural transitions were identified as windows whose first and second halves have substantially statistical different reactivity scores by the Wilcoxon test.

### Comparison analysis of structural information retrieval from DRS data

We performed a comprehensive benchmark comparison of DeCoRE against three existing methods (PORE-cupine, nanoSHAPE, and Nanocompore) using DRS data from 150 mM Aclm-treated pri-miR-17∼92a RNA. Evaluation was performed by calculating the AUC of the structural profile generated by different methods. Simply, the known RNA secondary structure is represented in dot-bracket notation, where each nucleotide is annotated as unpaired (“.”) or paired [“(” and “)”]. We concatenate the six elements of pri-miR-17∼92a RNA as a whole while simultaneously concatenating their corresponding scores. In the evaluation, nucleotides labeled as unpaired (“.”) are treated as the positive class (true), while paired nucleotides [“(” and “)”] are treated as the negative class. The predicted reactivity scores are then used to compute the AUC by assessing their ability to distinguish between paired and unpaired states. We used the provided structural profile in the original publication for nanoSHAPE, while the structural profile of the other two methods was computed using their default parameters using raw data from DRS as input.

### Comparison with DaVinci algorithm for structural deconvolution from DRS data

We conducted a focused comparison between DeCoRE and the DaVinci algorithm on DRS data of the lysine riboswitch and TPP riboswitch. The DaVinci algorithm (*17*) was applied to the vectorized structural profile of each read without miscalled bases information. The secondary structure model of the representative centroid structure for each cluster was extracted from the corresponding fold profiles to evaluate its agreement between the known riboswitches’ structures.

### Ethics statement

This research did not involve any experiments with human participants or live animals.
