# Supplementary material for: DeCoRE: A computational method to resolve RNA structural heterogeneity from RNA structure probing data by direct RNA sequencing
Source: Sci Adv. 2026 Oct 2;12(40):eaef0670. doi: 10.1126/sciadv.aef0670 (PMC13632732; doi:10.1126/sciadv.aef0670)
Supplement: Supplementary file 1 [file sciadv.aef0670_sm.pdf]

Science Advances
AAAS

Supplementary Materials for

DeCoRE: A computational method to resolve RNA structural heterogeneity from RNA structure probing data by direct RNA sequencing

Yong Huang *et al.*

Corresponding author: Lei Sun, sunlei0227@sdu.edu.cn; Qiangfeng Cliff Zhang, qczhang@tsinghua.edu.cn

Sci. Adv. **12**, eaef0670 (2026)
DOI: 10.1126/sciadv.aef0670

This PDF file includes:

Figs. S1 to S5
Table S1

Supplementary Figure 1

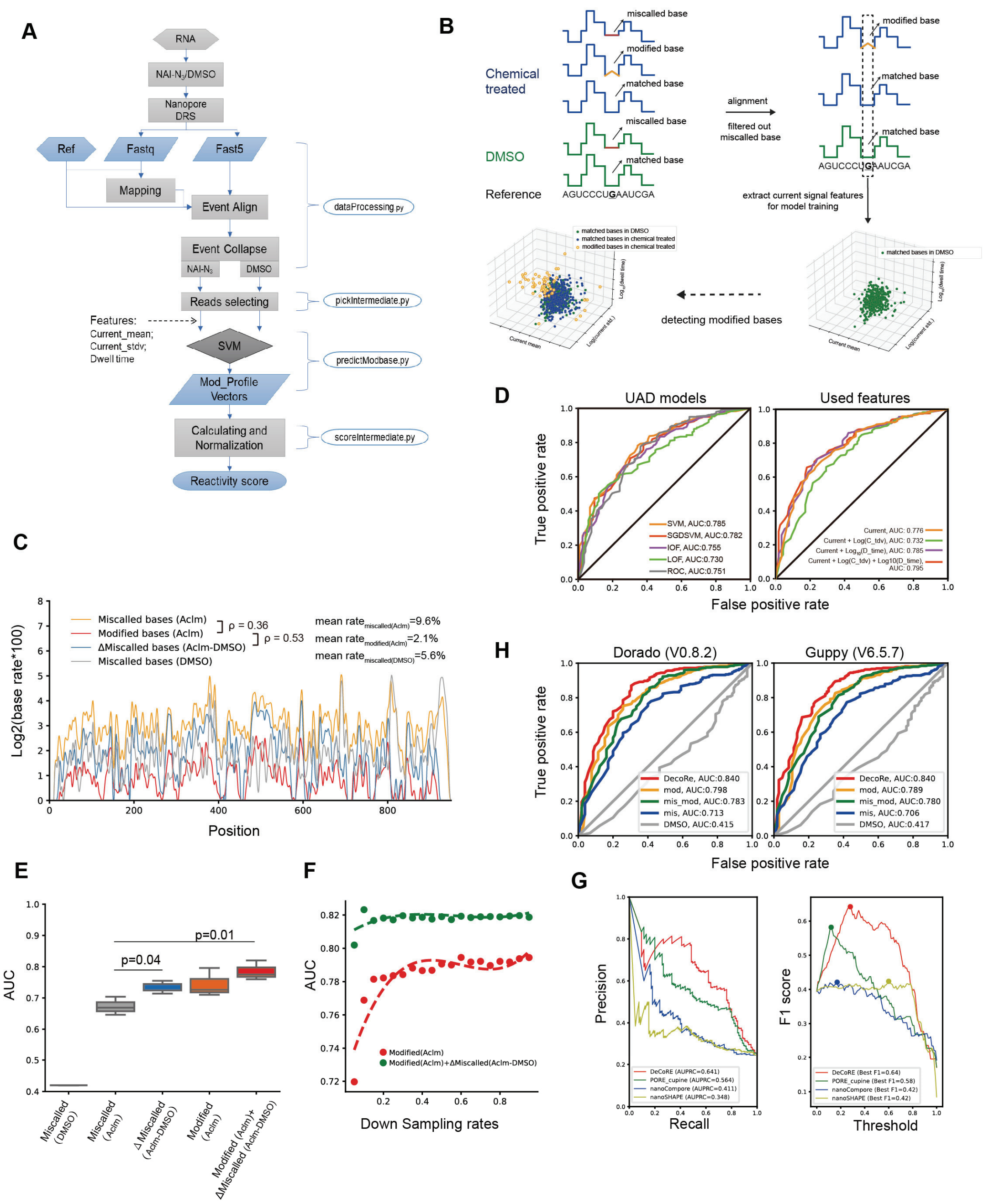

Fig. S1.

Establish the DeCoRE method through enhanced structural signal detection and machine-learning. (**A**) Schematic workflow of the DeCoRE computational pipeline, comprising four modular components, each implemented as an individual Python script. The dataProcessing script perform preprocessing of raw nanopore sequencing data by aligning current signal events from each mapped read to reference RNA, producing a clean event file. The pickIntermediate

script clusters reads according to their mapped start and end positions to reference RNA, effectively partitioning them into discrete transcriptional intermediates. This part could be skipped if users don't want to specific the intermediate. The predictModbase script detect the structural signals—including modified, miscalled and matched bases—within chemical treated reads, annotating each base with modification labels and transforming each read into a digital vector. The scoreIntermediate script calculates reactivity scores for each classified heterogeneous structure. (**B**) Using raw current signal, basecalled residues in chemical treated reads are categorized into three types: matched, miscalled, and modified (with an asterisk indicating a matched base exhibiting a shifted signal). In contrast, DMSO-treated reads contain only matched and miscalled bases. Typically, miscalled bases are discarded after alignment. The current signal features of matched bases in the control sample are used to train a model, which is subsequently applied to identify modified bases. (**C**) The miscalled and modified base rate of Aclm-treated and DMSO-treated samples. The mean base rates were calculated with all sites of the RNA. The y-axis represents the log2 -transformed rate of affected bases. (**D**) ROC curves of structural profile calculated using five UAD models(left). The input current signal features are Current_mean and Dwell time. ROC curves of structural profile calculate using 3 feature combinations on SVM model (right). (**E**)The AUC scores incorporating both miscalled bases and modified bases in 75 mM, 100mM and 150nMAclm-treated RNA, evaluated against known structural models of pri-miR-17~92a RNA. (**F**) The structural prediction performance across different coverage levels of input reads. The reads are those used in Fig. 1B with total number of 7,361. (**G**) Dorado (V0.8.2) and Guppy (V6.5.7) were used to basecall the raw fast5 files and perform the performance evaluation of structural profile as in Fig. 1B. (**H**) AUPRC curves and threshold-based F1 score of reactivity scores calculated across four methods.

Supplementary Figure 2

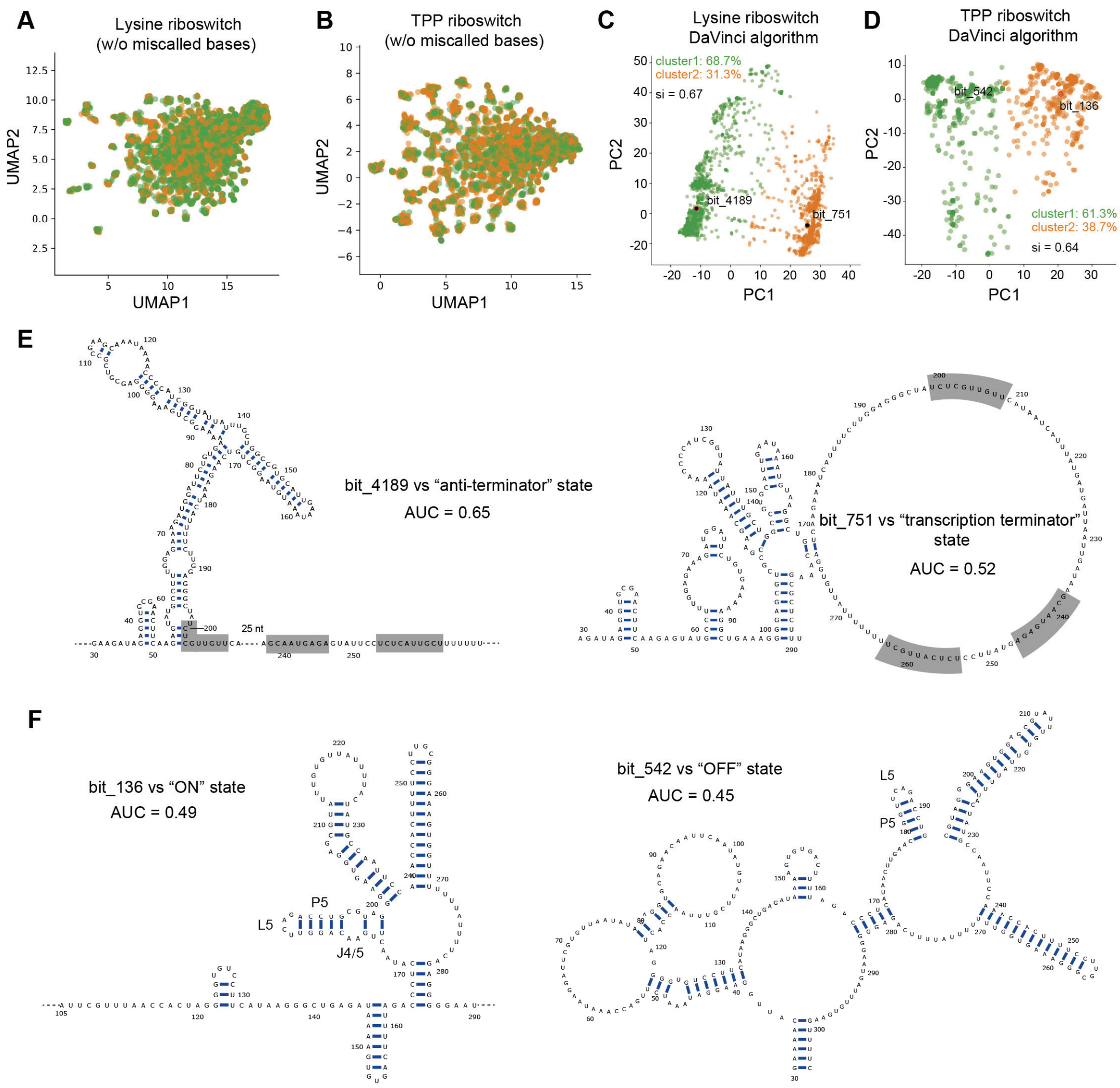

Fig. S2.

DeCoRE enhances clustering of heterogenous RNAs by harnessing the miscalled bases. (**A, B**) UMAP of the vectorized miscalled-base excluded structural profile of each read in chemical treated samples of the lysine riboswitch (A) and the TPP riboswitch (B). The points are colored by the cluster assignments in Fig. 2B and 2E. (**C, D**) Structural conformations of the lysine riboswitch (A) and the TPP riboswitch (B) inferred by the DaVinci algorithm, with clusters distinguished by color. Data were visualized using principal component analysis (PCA). Each dot represents a unique single structure derived from each single-molecule mutational profile by DaVinci algorithm. The representative centroid structures for each cluster were highlighted. (**E, F**) Structural models of the representative centroid structures of the two cluster of the lysine riboswitch (E) and the TPP riboswitch (F). The agreement between the predicted structural models and known structures were evaluated using AUC.

Supplementary Figure 3

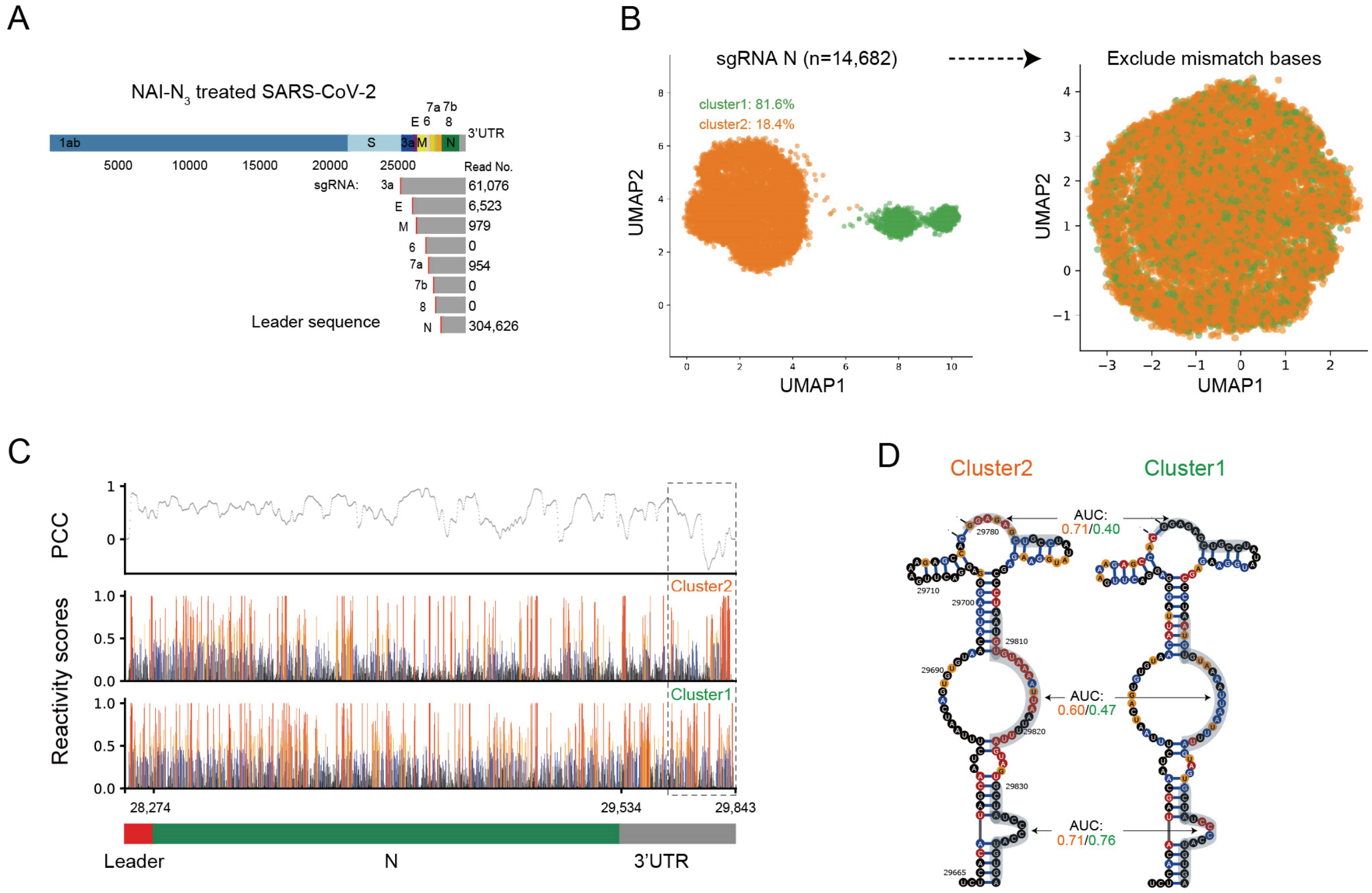

Fig. S3.
DeCoRE identifies RNA structural heterogeneity in a public SARS-CoV-2 RNA dataset. (**A**) The genomic structure of subgenomic RNAs of SARS-CoV-2, labelled with their corresponding mapped read counts. (**B**) The reads of sgRNA N were randomly down sampled for both two datasets and used for Modification bases detection and Clustering using UMAP. Then mismatch bases exclusion was applied to the data and perform Clustering again, resulting in the collapse of all reads into a single cluster. (**C**) The PCC of the reactivity scores between the two structural clusters of sgRNA N. (**D**) The calculated reactivity scores of each cluster were aligned to the reported average structure model of SARS-CoV-2 3'UTR. The AUC was calculated based on the local structure elements using the bases in gray boxes.

Supplementary Figure 4

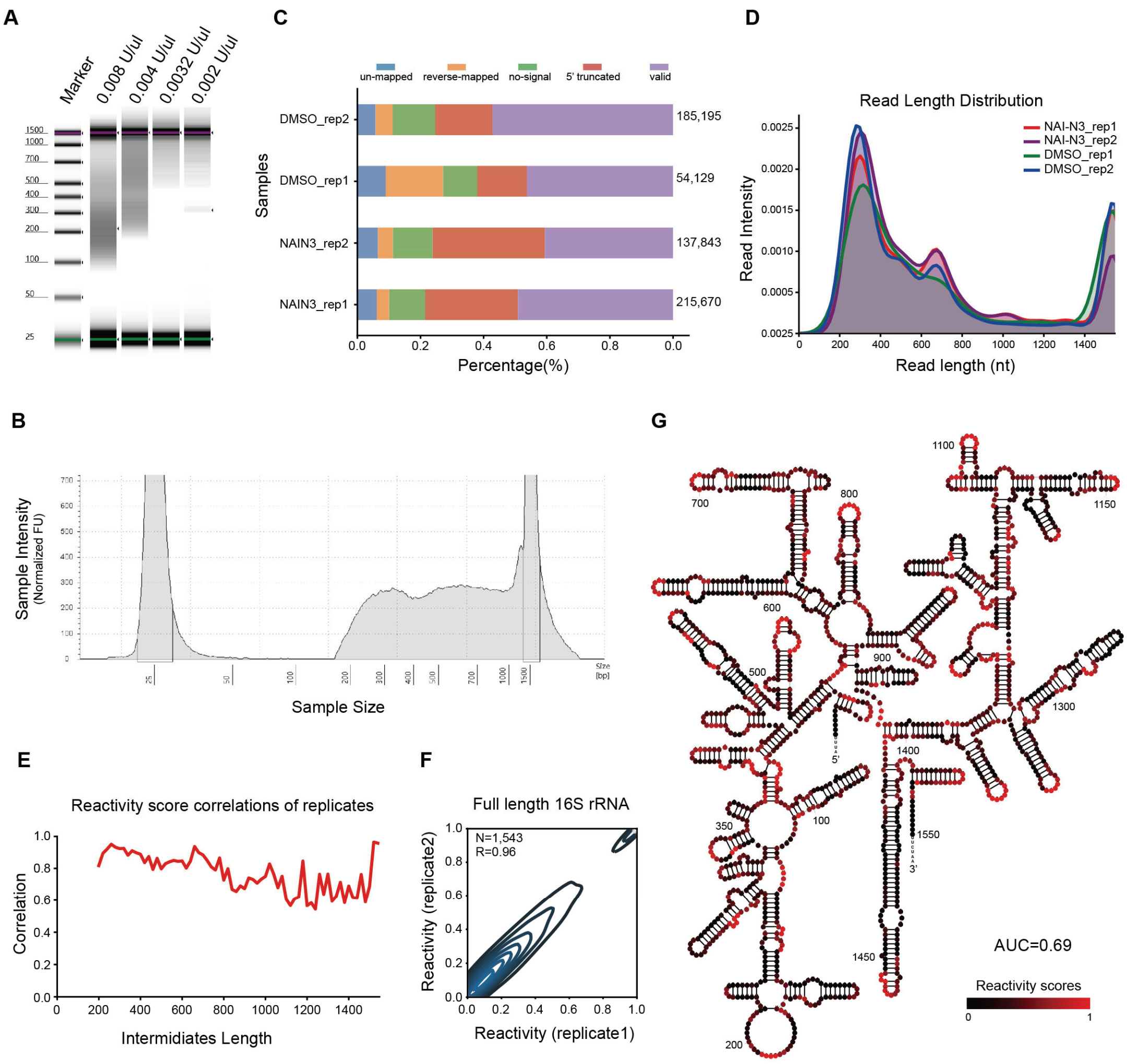

Fig. S4.

Quality assessment of nanopore DRS-based structure probing of the transcriptional intermediates of B. subtilis 16S rRNA. (**A**) Optimization of DNase I digestion conditions for template DNA preparation. Four DNase I concentrations were tested to optimize digestion efficiency, with 0.004U/ul identified as the optimal condition. (**B**) Length distribution of RNA transcripts generated by in vitro transcription. (**C**) Composition analysis of raw nanopore sequencing reads after quality control. After removal of unmapped, reverse-mapped, and 5’ truncated reads, 40–57% of reads were retained for analysis. (**D**) Length distribution of the sequencing reads from both NAI-N3- and DMSO-treated replicates. (**E**) Inter-replicate reproducibility assessment. PCCs of reactivity scores between two biological replicates for each intermediate. (**F**) Replicate correlation analysis. Scatterplot showing correlation of the reactivity scores of full-length 16S rRNA between replicates. (**G**) The known structural model of B. subtilis 16S rRNA, colored by reactivity scores of full-length 16S rRNA.

Supplementary Figure 5

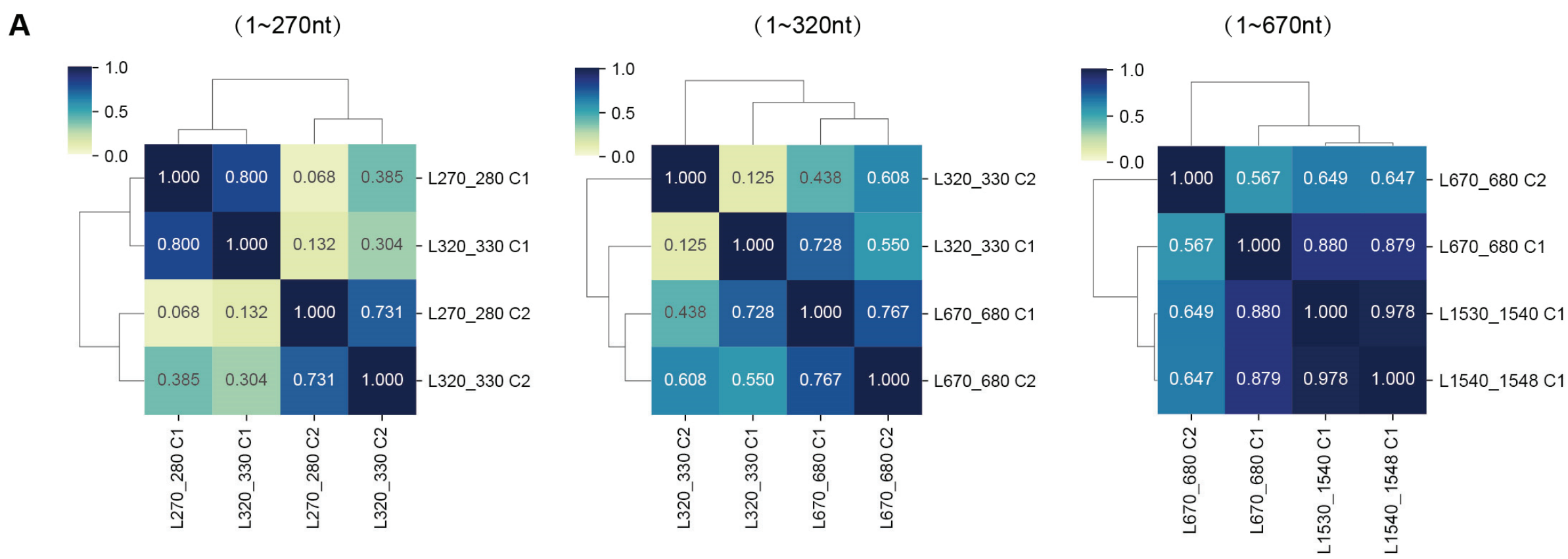

Fig. S5.

Structural heterogeneity and transition of 16S rRNA during transcription. (**A**) Pairwise structural similarity analysis of adjacent transcriptional intermediates. Heatmaps display PCC of reactivity scores between adjacent transcriptional intermediates, calculated over their shared regions (demarcated above each heatmap).

Table S1.

Comparison of existing methods for retrieval of structural information from nanopore DRS-based RNA structure probing data.

Methods	Modification detection	Used Features	Available tool	Mod Profiling*
PORE-cupine	SVM model	Current_mean Current_stdv	R code https://github.com/awjga/PORE-cupine	Yes
nanoSHAPE	KS test	Current_mean or Dwell time	Recodes https://github.com/physnano/rRNA_nanoSHAPE	No
Nanocompore	Gaussian Mixture model or KS test	Current_mean Dwell time	Python code https://nanocompore.rna.rocks/	No
DeCoRE	SVM model	Current_mean Dwell time Current_stdv	Python code https://github.com/huangyon20/DeCoRE	Yes

*: If each base of the reads could be labeled with the modification status.
